# Transgenic miR156 switchgrass in the field: growth, recalcitrance and rust susceptibility

**DOI:** 10.1111/pbi.12747

**Published:** 2017-06-20

**Authors:** Holly L. Baxter, Mitra Mazarei, Alexandru Dumitrache, Jace M. Natzke, Miguel Rodriguez, Jiqing Gou, Chunxiang Fu, Robert W. Sykes, Geoffrey B. Turner, Mark F. Davis, Steven D. Brown, Brian H. Davison, Zeng‐Yu Wang, C. Neal Stewart

**Affiliations:** ^1^ Department of Plant Sciences University of Tennessee Knoxville TN USA; ^2^ BioEnergy Science Center (BESC) Oak Ridge National Laboratory Oak Ridge TN USA; ^3^ Biosciences Division Oak Ridge National Laboratory Oak Ridge TN USA; ^4^ Samuel Roberts Noble Foundation Ardmore OK USA; ^5^ National Renewable Energy Laboratory Golden CO USA

**Keywords:** microRNA156, biomass, transgene regulation, switchgrass, flowering, bioconfinement

## Abstract

Sustainable utilization of lignocellulosic perennial grass feedstocks will be enabled by high biomass production and optimized cell wall chemistry for efficient conversion into biofuels. MicroRNAs are regulatory elements that modulate the expression of genes involved in various biological functions in plants, including growth and development. In greenhouse studies, overexpressing a microRNA (*miR156*) gene in switchgrass had dramatic effects on plant architecture and flowering, which appeared to be driven by transgene expression levels. High expressing lines were extremely dwarfed, whereas low and moderate‐expressing lines had higher biomass yields, improved sugar release and delayed flowering. Four lines with moderate or low miR156 overexpression from the prior greenhouse study were selected for a field experiment to assess the relationship between miR156 expression and biomass production over three years. We also analysed important bioenergy feedstock traits such as flowering, disease resistance, cell wall chemistry and biofuel production. Phenotypes of the transgenic lines were inconsistent between the greenhouse and the field as well as among different field growing seasons. One low expressing transgenic line consistently produced more biomass (25%–56%) than the control across all three seasons, which translated to the production of 30% more biofuel per plant during the final season. The other three transgenic lines produced less biomass than the control by the final season, and the two lines with moderate expression levels also exhibited altered disease susceptibilities. Results of this study emphasize the importance of performing multiyear field studies for plants with altered regulatory transgenes that target plant growth and development.

## Introduction

Developing alternative energy sources is imperative for enhancing energy security and offsetting greenhouse gas emissions associated with the use of fossil fuels. Lignocellulosic biomass represents an abundant and carbon‐neutral nonfood source of plant material that could be exploited for the sustainable production of biofuels. One attractive candidate for a dedicated lignocellulosic feedstock is switchgrass (*Panicum virgatum* L.), a C4 perennial bunchgrass native to the United States. Like many other C4 perennial grasses, switchgrass can produce high amounts of biomass with relatively low agricultural inputs owing to its high water and nutrient use efficiency (van der Weijde *et al*., 2013). Furthermore, switchgrass has a wide geographic distribution across North America and appears to be adaptable to diverse environmental conditions and soil types (Parrish and Fike, 2005). However, switchgrass and other lignocellulosic feedstocks have evolved structurally complex and heterogeneous cell walls that are intrinsically resistant, or recalcitrant, to existing chemical and biological deconstruction methods (DeMartini *et al*., 2013). Secondary cell walls of plants consist of a cross‐linked matrix of polysaccharides (cellulose and hemicelluloses), lignin and minor amounts of structural proteins (Pauly and Keegstra, 2008). Biomass recalcitrance is influenced by the relative amounts of these components as well as their interactions with one another (Zhao *et al*., 2012). Cost‐effective production of fuels from dedicated lignocellulosic grasses will require maximizing biomass productivity per unit of land and optimizing the cell wall structure for more efficient conversion into fuels (van der Weijde *et al*., 2013), both of which have been central goals of biotechnology in the bioenergy field.

Genetic engineering of plant cell walls for improved biofuel production often involves modifying cell wall properties that contribute to recalcitrance, such as the cell wall lignin content (Cai *et al*., 2016; Fornalé *et al*., 2012; Fu *et al*., 2011a,b; Jung *et al*., 2012; Poovaiah *et al*., 2016; Saathoff *et al*., 2011; Shen *et al*., 2012, 2013; Xu *et al*., 2011). Other equally important targets for improvement are biomass yield and plant architecture (Mauro‐Herrera and Doust, 2016; Stamm *et al*., 2012), both of which have recently been modified in switchgrass (Poovaiah *et al*., 2015; Wuddineh *et al*., 2015). However, targeting yield‐related traits can be challenging as they are often controlled by diverse genes from different genetic pathways and are also strongly influenced by environment. An attractive approach for manipulating such complex traits is through the modification of master regulators that have multiple gene targets involved in various aspects of plant growth and development. microRNAs (miRNAs) are an important class of gene regulatory factors that affect several plant processes including auxin signalling (Mallory *et al*., 2005), juvenile‐to‐adult growth transition (Poethig, 2013), floral organ identity and flowering time (Mallory *et al*., 2004), and environmental stress responses (Liu *et al*., 2008; Navarro *et al*., 2006) and have recently been recognized for their potential for trait improvement in biofuel feedstocks (Trumbo *et al*., 2015).

In particular, overexpression of miRNAs from the miR156 family, which target *SQUAMOSA PROMOTOR BINDING PROTEIN‐LIKE* (*SPL*) transcription factor genes have demonstrated significant potential for improving biofuel‐related traits in important bioenergy species (Chuck *et al*., 2011; Fu *et al*., 2012; Rubinelli *et al*., 2013). Plants undergo a vegetative phase of growth, during which there is a rapid accumulation of biomass and an increase in size, followed by a transition to reproductive growth and seed set (Poethig, 2013). Prolonging the vegetative growth stage by delaying or eliminating flowering is desirable as it could allow for a greater accumulation of biomass. miR156, which regulates the vegetative‐to‐reproductive phase change in several plant species, is present in high levels during the juvenile phase of growth and then declines after the transition to reproductive growth (Chuck *et al*., 2007; Wu and Poethig, 2006; Wu *et al*., 2009). Overexpression of the maize *miR156 (Corngrass1*) gene in switchgrass prolonged the vegetative phase and eliminated flowering under greenhouse and field conditions (Chuck *et al*., 2011). These plants were also more digestible and released more glucose during saccharification assays. Similarly, overexpression of the rice OsmiR156b gene precursor in switchgrass resulted in a reduction or complete elimination of flowering in several lines and enhanced biomass yields by up to 101% over the control (Fu *et al*., 2012). From a bioconfinement standpoint, inhibition of flowering would be a valuable trait in transgenic biofuel feedstocks for preventing transgene flow into native wild plant populations (Kausch *et al*., 2010).

In the study by Fu *et al*. (2012), miR156 overexpression in greenhouse‐grown switchgrass resulted in a broad range of morphological modifications that corresponded to the relative level of miR156 overexpression. Expression levels of several miR156‐targested switchgrass *SPL* (*PvSPL*) genes were also shown to be suppressed by varying degrees among the transgenic lines. Accordingly, the transgenic lines were categorized into three groups with low, moderate, or high expression levels of miR156. Those with relatively low miR156 levels had increased tillering and higher biomass yield than the control and flowered normally. Moderate levels of miR156 resulted in shorter plants with more tillers and higher biomass production, while those with the highest levels of expression were severely dwarfed and produced substantially less biomass than the control. Transgenic lines with moderate and high levels of miR156 did not flower for the duration of the greenhouse study. Some lines also had improved sugar release depending on whether or not a pretreatment was performed (Fu *et al*., 2012).

Assessing growth in the field is necessary validation step for genetically engineered plants as it is well‐known that initial greenhouse observations are not always predictive of long‐term field performance. Validation under field conditions is especially important in plants overexpressing transgenes that influence yield and yield‐related traits, as prior studies have shown inconsistencies in growth phenotypes between greenhouse and field environments (Viswanath *et al*., 2011; Voorend *et al*., 2015). Extensive field testing may reveal downstream effects of genetic modifications that might not be apparent in a tightly controlled greenhouse environment. Another limitation of greenhouse studies is the inability to assess potential impacts of transgenic modifications on disease susceptibility. In the present study, four promising miR156‐overexpressing transgenic lines from the greenhouse study were selected for further analysis under field conditions. Included were two lines with low expression levels (T14 and T35) and two lines with moderate expression levels (T27 and T37) (Fu *et al*., 2012). These plants were analysed over the course of three field growing seasons for (i) *miR156* and *PvSPL* gene expression, (ii) growth morphology and biomass yield, (iii) cell wall chemistry and bioconversion efficiency and (iv) susceptibility to switchgrass rust (*Puccinia emaculata*).

## Results

### *miR156* gene expression

**Figure 1:**
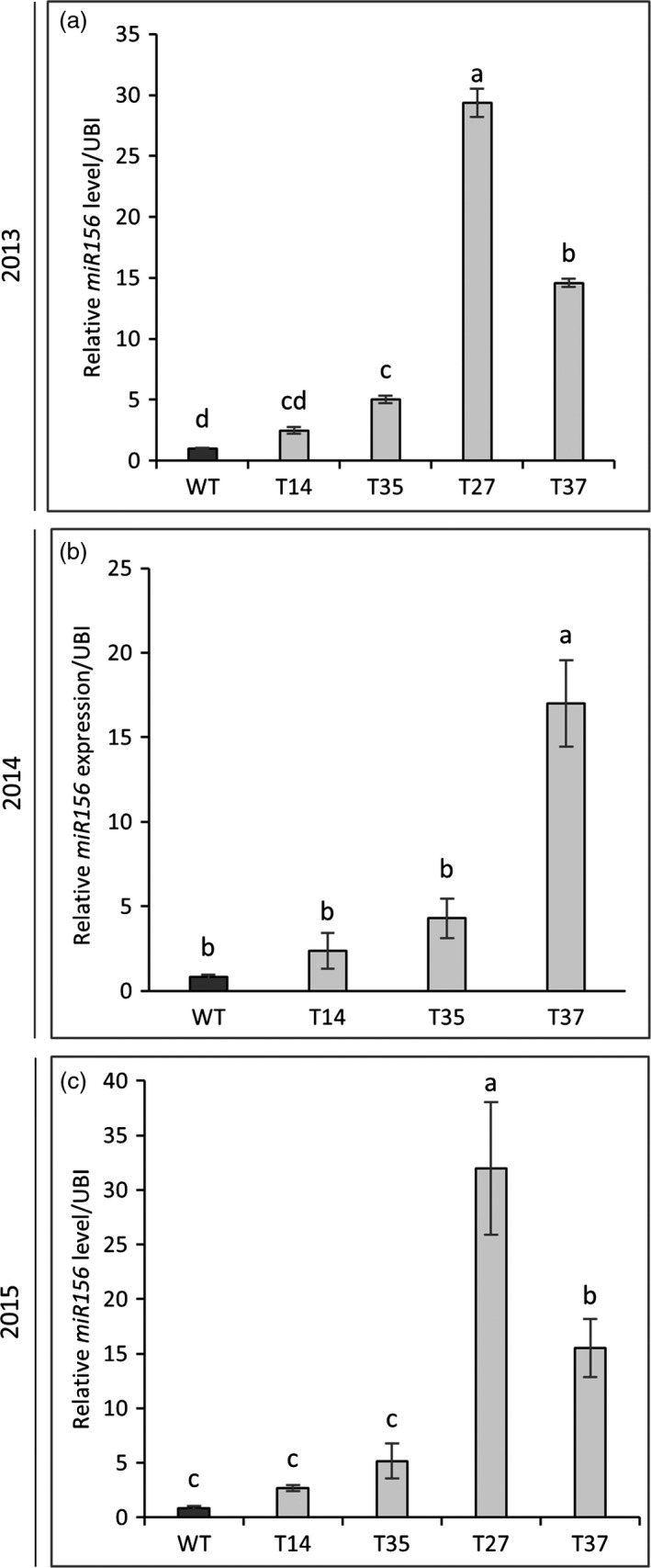
Expression level of mature *miR156* in R1‐stage tillers of transgenic switchgrass plants as determined by quantitative RT‐PCR. Samples were harvested in years one (a), two (b) and three (c) of the field trial. The relative levels of transcripts were normalized to ubiquitin (UBI). Bars represent the mean of the biological replicates (*n* = 4) for each transgenic line (T14, T35, T27, T37) and the wild‐type control (WT) ± standard error. Means within each year were compared with a one‐way ANOVA and letter groupings were obtained using Fisher's least significant difference method. Bars with different letters are significantly different at the 5% level.

To confirm that the transgene was retained and expressed in the field‐grown miR156‐overexpressing switchgrass lines, expression levels of the rice OsmiR156b precursor in samples harvested during year one (2013) were measured by qRT‐PCR. OsmiR156b was detected in all transgenic lines, with the highest level of expression occurring in line T27 (Figure S1). Mature switchgrass miR156 levels were also measured in the transgenic lines and the control each year. Expression patterns were consistent among the three growing seasons. In line with the greenhouse findings (Fu *et al*., 2012), miR156 expression in lines T14 and T35 was relatively low, whereas expression was significantly higher in lines T27 and T37 (Figure 1). miR156 expression in line T37 was increased by 15‐ to 21‐fold over the control across the 3‐year study. The highest level of miR156 expression was observed in line T27, which exhibited a 29‐ to 37‐fold increase over the control in years one and three (Figure 1). These results are in line with the analysis of OsmiR156b expression, for which T27 showed a significantly higher transcript abundance relative to the other transgenic lines (Figure S1).

### *PvSPL* gene expression

To evaluate the effect of miR156 overexpression on switchgrass SPL genes, expression levels of PvSPL1, PvSPL2, PvSPL3 and PvSPL6 were measured on green tissue harvested in mid‐season of years one (2013) and three (2015). In year one (2013), PvSPL1 levels were reduced by twofold in the low miR156‐expressing transgenic lines (T14 and T35) and three‐ to fourfold in the moderate miR156‐expressing transgenic lines (T27 and T37) relative to the control (Figure S2a). Similarly, PvSPL6 levels were reduced by two‐ to threefold in the low‐expressing lines and 10‐ to 15‐fold in the moderate‐expressing lines (Figure S2d). PvSPL2 levels were reduced by one‐ to twofold in all lines except for T14, while PvSPL3 was only reduced (twofold decrease) in line T27 (Figure S2b, c).

In year three, PvSPL2 levels were reduced by one‐ to twofold in the low miR156‐expressing lines (T14 and T35), and three‐ to fivefold in the moderate miR156‐expressing lines (T27 and T37) relative to the control (Figure S3b). Similar to year one, PvSPL6 expression levels were reduced by two‐ to threefold in low‐expressing lines and seven‐ to 10‐fold in moderate‐expressing lines (Figure S3d). Expression levels of PvSPL1 and PvSPL3 were reduced by two‐ to fivefold in all lines except for T35 (Figure S3a, c).

### Growth phenotypes and flowering

Plant growth was evaluated and flowering was monitored across each growing season. Flowering was observed in all transgenic lines except for T27, which never flowered for the duration of the experiment. Transgenic lines exhibited a wide range of growth phenotypes across the 2 years, which were generally consistent between mid‐season (Table S1) and end‐of‐season (Table 1) within each year. By the end of the establishment year (2013), T35 plants were 18% taller and 22% wider than the wild‐type control. T37 was 37% taller and produced 100% more tillers than the control. Line T14 was similar to the control in height and tiller number, but had a 32% decrease in width (Table 1). Line T27 was 30% shorter than the control but produced 430% more tillers (Table 1). Biomass yield was increased by 56% in line T35 and 149% in line T37 relative to the control. Despite the reduction in height, T27 produced an equivalent amount of biomass as the control resulting from the increased number of tillers. Line T14 exhibited a 44% reduction in biomass yield relative to the control (Table 1).

**Table 1:** End‐of‐season morphology and ethanol yield of miR156‐overexpressing switchgrass in the first (2013), second (2014) and third (2015) field growing seasons

Year	Line	Tiller height (cm)	Plant width (cm)	Tiller number	Dry weight yield (g/plant)	Ethanol yield (mg/g biomass)	Total ethanol (mg/plant)*
2013	T14	84.8 ± 3.1^c^	99.2 ± 3.2^d^	50.6 ± 2.7^c^	68.5 ± 3.5^d^	nd	
	T35	113.0 ± 5.5^b^	177.3 ± 7.9^a^	70.9 ± 7.0^c^	190.6 ± 11.4^b^	43.0 ± 0.9^a^	8195.80
	T27	66.7 ± 2.9^d^	138.7 ± 8.3^c^	326.1 ± 53.2^a^	135.9 ± 17.5^c^	nd	
	T37	130.5 ± 5.2^a^	161.9 ± 3.6^ab^	123.7 ± 25.5^b^	303.4 ± 18.5^a^	42.7 ± 1.9^a^	12955.18
	WT	95.4 ± 5.0^c^	145.3 ± 11.5^bc^	61.6 ± 13.0^c^	121.9 ± 18.5^c^	36.4 ± 1.7^b^	4437.16
2014	T14	154.3 ± 4.3^b^	187.0 ± 9.9^d^	149.0 ± 11.9^d^	626.4 ± 64.7^c^	nd	
	T35	172.8 ± 4.7^a^	297.2 ± 11.0^a^	252.8 ± 15.8^bc^	1244.6 ± 82.1^a^	27.4 ± 1.3^a^	34102.04
	T27	67.9 ± 0.9^c^	229.6 ± 4.1^bc^	777.3 ± 45.4^a^	202.6 ± 23.9^d^	nd	
	T37	179.1 ± 2.9^a^	211.1 ± 3.6^c^	320.6 ± 21.5^b^	968.0 ± 70.4^b^	25.7 ± 2.6^a^	24877.60
	WT	171.6 ± 3.9^a^	249.7 ± 3.8^b^	206.9 ± 7.3^cd^	999.1 ± 38.3^b^	32.7 ± 3.3^a^	32670.57
2015	T14	171.5 ± 4.6^c^	232.7 ± 0.9^c^	236.3 ± 9.0^a^	1035.6 ± 65.0^c^	nd	
	T35	204.3 ± 2.1^a^	367.8 ± 1.7^a^	246.4 ± 12.1^a^	1825.6 ± 111.3^a^	17.9 ± 1.9^a^	32678.24
	T27	68.7 ± 1.9^d^	134.9 ± 16.3^e^	153.3 ± 44.0^a^	52.5 ± 16.3^d^	nd	
	T37	192.2 ± 1.5^b^	205.1 ± 3.1^d^	241.4 ± 10.7^a^	850.6 ± 42.8^c^	15.7 ± 0.7^a^	13355.99
	WT	195.0 ± 0.8^b^	297.5 ± 4.9^b^	218.1 ± 12.6^a^	1451.9 ± 60.3^b^	17.0 ± 0.7^a^	24682.30

Values represent the mean of the biological replicates (*n* = 4) for each transgenic line (T14, T35, T27, T37) and the wild‐type control (WT) ± standard error. Means within each year were compared with a one‐way ANOVA, and letter groupings were obtained using Fisher's least significant difference method. Values followed by different letters are significantly different at the 5% level. Nd, not determined.

*Total ethanol (mg/plant) was calculated by multiplying the ethanol yield (mg/g biomass) by the dry weight yield (g/plant).

In December of the subsequent season (2014), line T35 continued to exhibit an increased plant width (19%) but was similar to the control in tiller height. T37 was similar to the control in height and produced 55% more tillers, but exhibited a 15% reduction in width. A reduction in height (10%) and width (25%) was observed in line T14. T27 produced 275% more tillers than the control, but had a 60% reduction in height. Biomass yield was increased by 25% in line T35, and unchanged in line T37. Line T27, despite an increase in tiller number, exhibited a yield reduction of 80% relative to the control. Line T14 exhibited a yield reduction of 37% relative to the control (Table 1).

By the end of the third growing season (2015), T35 was 5% taller, 24% wider, and exhibited a similar number of tillers as the control. As in the previous growing season (2014), line T37 was similar to the control in height with a reduction in width (31%), but no increase in tiller number was observed. In line T14, height was decreased by 12%, width was decreased by 22% and tiller number was unchanged relative to the control. Similarly, T27 showed a 65% reduction in height and 55% reduction in width, with no change in tiller number relative to the control. T35 continued to produce significantly more biomass than the control, with a yield improvement of 26%. Lines T14, T27 and T37 showed yield reductions of 29%, 96% and 41%, respectively, compared to the control (Table 1).

### Lignin content and S/G ratio

Cell wall lignin content and the S/G ratio were determined for green reproductive‐stage tillers harvested at mid‐growing season and mature senesced biomass harvested at the end of the season. In mid‐season of year one (2013), lignin was increased by 8% in lines T14, T27 and T37, and unchanged in line T35. T27 also exhibited 9% decrease in the S/G ratio (Table S2). After senescence, all transgenic lines had similar lignin levels as the control but S/G ratios were variable: lines T27 and T37 showed decreases of 9%–16%, while an increase of 7% was observed in T14 (Table 2). In the second year (2014), all lines had similar lignin content as the control except for T27, which showed a 9% decrease after senescence. S/G ratios were reduced by 14%–21% in green and senesced material from T27 and T37. Line T35 also exhibited a decrease (5%) in the S/G ratio but only after senescence. The S/G ratio of T14 was unchanged across the season (Tables 2 and S2). In the third year (2015), a 4% decrease in lignin content was observed in mid‐season material from line T37, and a 7%–10% decrease in lignin content was observed in mid‐ and end‐of‐season material from T27. S/G ratios in these two lines were decreased by 14%–21% across the season. The lignin contents and S/G ratios of lines T14 and T35 were unchanged relative to the control at both green and senesced stages (Tables 2 and S2).

**Table 2:** Cell wall characterization of miR156‐overexpressing switchgrass harvested at the end of the growing season in years one (2013), two (2014) and three (2015) of the field experiment

Year	Line	Lignin content (% CWR)	S/G ratio	Glucose release (mg/g CWR)	Xylose release (mg/g CWR)	Total sugar release (g/g CWR)
2013	T14	22.1 ± 0.3^a^	0.64 ± 0.01^a^	0.209 ± 0.01^a^	0.190 ± 0.01^a^	0.399 ± 0.01^a^
	T35	22.0 ± 0.4^a^	0.63 ± 0.01^ab^	0.211 ± 0.01^a^	0.191 ± 0.01^a^	0.402 ± 0.01^a^
	T27	22.3 ± 0.3^a^	0.50 ± 0.01^d^	0.204 ± 0.01^a^	0.199 ± 0.01^a^	0.403 ± 0.01^a^
	T37	21.5 ± 0.5^a^	0.54 ± 0.02^c^	0.209 ± 0.01^a^	0.198 ± 0.01^a^	0.406 ± 0.01^a^
	WT	20.9 ± 0.1^a^	0.60 ± 0.01^b^	0.209 ± 0.01^a^	0.181 ± 0.01^a^	0.390 ± 0.01^a^
2014	T14	24.7 ± 0.1^a^	0.65 ± 0.01^a^	0.149 ± 0.01^d^	0.184 ± 0.01^c^	0.332 ± 0.01^d^
	T35	23.8 ± 0.4^ab^	0.64 ± 0.01^a^	0.178 ± 0.01^bc^	0.191 ± 0.01^bc^	0.369 ± 0.01^bc^
	T27	21.4 ± 0.5^c^	0.51 ± 0.01^b^	0.199 ± 0.01^a^	0.199 ± 0.01^ab^	0.398 ± 0.01^a^
	T37	23.1 ± 0.5^b^	0.54 ± 0.01^b^	0.180 ± 0.01^c^	0.209 ± 0.01^a^	0.389 ± 0.01^ab^
	WT	23.6 ± 0.5^ab^	0.63 ± 0.01^a^	0.163 ± 0.01^cd^	0.191 ± 0.01^bc^	0.354 ± 0.01^cd^
2015	T14	24.3 ± 0.2^a^	0.65 ± 0.00^a^	0.117 ± 0.01^a^	0.156 ± 0.01^a^	0.273 ± 0.01^a^
	T35	23.9 ± 0.4^a^	0.66 ± 0.01^a^	0.116 ± 0.01^a^	0.150 ± 0.01^a^	0.266 ± 0.02^a^
	T27	21.9 ± 0.1^b^	0.53 ± 0.00^c^	0.159 ± 0.01^a^	0.185 ± 0.01^a^	0.344 ± 0.02^a^
	T37	23.4 ± 0.5^a^	0.57 ± 0.01^b^	0.135 ± 0.01^a^	0.183 ± 0.01^a^	0.318 ± 0.02^a^
	WT	23.5 ± 0.4^a^	0.67 ± 0.03^a^	0.127 ± 0.01^a^	0.171 ± 0.01^a^	0.297 ± 0.02^a^

Aboveground senesced biomass harvested at the end of the growing season was analysed for lignin content, syringyl‐to‐guaiacyl (S/G) lignin monomer ratio and sugar release by enzymatic hydrolysis. Values represent the mean of the biological replicates (*n* = 4) for each transgenic line (T14, T35, T27, T37) and the wild‐type control (WT) ± standard error. Means within each year were compared with a one‐way ANOVA, and letter groupings were obtained using Fisher's least significant difference method. Values followed by different letters are significantly different at the 5% level. CWR, cell wall residues.

### Sugar release and ethanol yield

Sugar release by enzymatic hydrolysis was measured on hot water‐pretreated (180 °C, 17.5 m) green and senesced material each season. In the first year (2013), lines T27 and T37 had a 12% lower sugar release relative to the control at mid‐season but were similar to the control after senescence. Sugar release was unchanged in T14 and T35 at both green and senesced stages (Tables 2 and S2). In the second year (2014), line T27 had a 12%–18% higher sugar release than the control prior to and after senescence. Line T37 also exhibited an increase in sugar release (10%) but only after senescence. Consistent with the first year, the sugar release of lines T14 and T35 was equivalent to the control (Tables 2 and S2). In the third year (2015), a 14% increase in sugar release was observed in lines T27 and T37 relative to the control at the green stage. After senescence, no changes in sugar release were observed among the transgenic and control lines (Tables 2 and S2).

Based on their overall superior growth performance, low miR156‐expressing line T35 and moderate‐miR156 expressing line T37 were selected for separate hydrolysis and fermentation (SHF) experiments to evaluate bioconversion efficiency. Both transgenic lines exhibited improvements (17%–18% increase) relative to the control in the first year, but no changes were observed in years to or three (Table 1).

### Disease susceptibility

**Figure 2:**
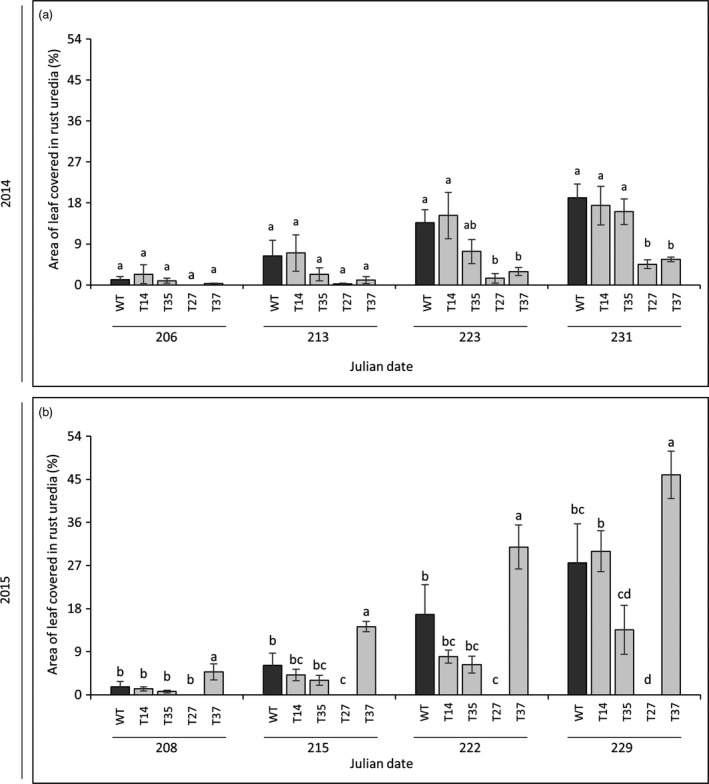
Susceptibility of miR156‐overexpressing switchgrass to rust (*P. emaculata*) in 2014 (a) and 2015 (b). Rust severity was determined as the percentage of the leaf surface covered in rust uredia. Bars represent the mean of the biological replicates (*n* = 4) for each transgenic line (T14, T35, T27, T37) and the wild‐type control (WT) ± standard error. For each year, means within each time point were compared with a one‐way ANOVA and letter groupings were obtained using Fisher's least significant difference method. Bars with different letters are significantly different at the 5% level.

**Figure 3:**
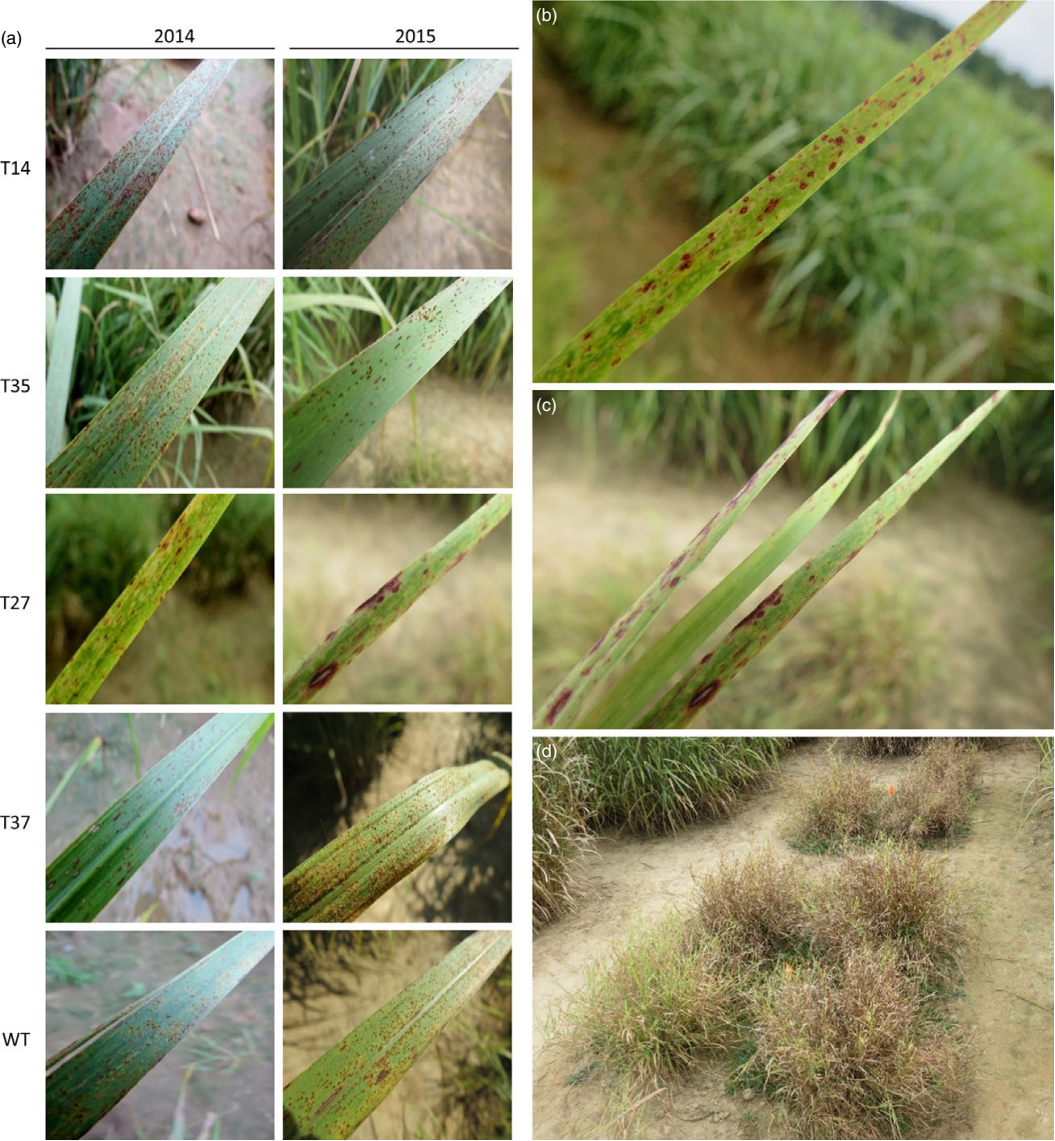
Photographs of fungal disease symptoms in miR156‐overexpressing switchgrass. (a) Rust symptoms caused by *P. emaculata* in each transgenic line (T14, T35, T27, T37) and the wild‐type control (WT) in 2014 and 2015. No rust was observed in line T27 in 2015; photograph shows *Bipolaris* leaf spot symptoms. (b, c) *Bipolaris* leaf spot symptoms in line T27 in (b) 2014 and (c) 2015. (d) Leaf damage and early browning in line T27 in September 2015.

Rust (*P. emaculata*) was the predominant pathogen affecting the switchgrass plants during the field experiment. The susceptibility of the transgenic lines to rust was rated on a weekly basis across a 4‐week period during the second (2014) and third (2015) growing seasons. Rust uredia appeared in late July and infection progressed significantly through late August of both growing seasons, after which the field was sprayed with a fungicide to prevent excessive damage to the plants. In 2014, the transgenic lines initially showed similar degrees of rust severity relative to the control. Differences became apparent during the last 2 weeks of rating, where lines T27 and T37 both showed significantly lower (71%–90%) susceptibilities relative to the control (Figures 2a and 3a). In the subsequent season (2015), line T27 showed no rust symptoms at any time points. Unlike the previous year, line T37 was more susceptible than the control across all time points, although the degree of difference between the two lines was reduced as rust progressed: rust severity was 195% higher in T37 relative to the control at the first time point, but only 67% higher after 4 weeks. As in the previous year, lines T14 and T35 showed similar levels of infection as the control across all time points in 2015 (Figures 2b and 3a).

Leaf spot caused by a *Bipolaris* species was also present among lines for the duration of the field trial. For most transgenic lines and the control, *Bipolaris* symptoms were minor. Line T27, however, appeared to be highly susceptible as demonstrated by the dark purple spots covering the surface of the leaves (Figure 3b, c). Progression of *Bipolaris* in this line might have contributed to the early browning and dying back of the severely infected leaves that was observed in late September of 2015 (Figure 3d).

## Discussion

There has been recent interest in discovering and manipulating genes involved in the vegetative‐to‐reproductive growth transition as a strategy to extend the vegetative phase of plant growth and increase biomass production. Overexpression of miR156 has been shown to prolong the vegetative phase and increase biomass yields in switchgrass (Chuck *et al*., 2011; Fu *et al*., 2012). In the present study, we evaluated the field performance of four low and moderate miR156‐overexpressing switchgrass lines selected from the previous greenhouse study (Fu *et al*., 2012). The 3‐year field study revealed inconsistencies in growth phenotypes between greenhouse and field‐grown transgenic lines, variability in growth and cell wall traits from season to season, and some negative effects on disease susceptibility in the higher‐expressing transgenic lines. Our results suggest a strong environmental influence on the growth and cell wall phenotype of miR156‐overexpressing transgenic switchgrass lines. This is the first multiyear field study examining a transgenic bioenergy feedstock genetically engineered with the goal of increasing biomass.

### Gene expression and growth

Relative miR156 expression levels in the field‐grown transgenic lines were consistent with the greenhouse observations (Fu *et al*., 2012): expression levels in T14 and T35 were relatively low, while levels in lines T27 and T37 were higher (Figure 1). Also in line with the greenhouse study, the expression of several *PvSPL* genes was suppressed among the field‐grown transgenic lines, with the greatest degree of suppression generally occurring in the two higher miR156‐overexpressing lines (T27 and T37) (Figure S2 and S3). *SPL* genes control several biological processes in plants including plant architecture (Preston and Hileman, 2013). A recent study linked the suppression of *PvSPL1* and *PvSPL2* genes, both of which are targeted by miR156, to a dramatic increase in tiller number in switchgrass (Wu *et al*., 2016). Accordingly, many of the greenhouse‐grown miR156‐overexpressing lines had lower levels of these two *PvSPL* genes and significant increases in tiller number relative to the control. In several of these lines, the increase in tiller number allowed them to compensate for unfavourable traits (reduced height, narrow leaves) and enabled higher biomass yields relative to the control (Fu *et al*., 2012).

Interestingly, the increased tillering phenotype was either absent (as observed in T14 and T35) or only present in the first two seasons (as observed in T27 and T37) in the field‐grown transgenic lines. Low‐expressing line T35 had sustained improvements in yield of up to 56% across the 3‐year experiment, which appeared to be associated with increased height and width rather than tiller number. Taking the yield increase into consideration, 30% more ethanol (mg/plant) could be expected from the third year harvest of T35 relative to the nontransgenic control (Table 1). However, the other low‐expressing transgenic line (T14) had reductions in height and width, thinner leaves and stems (Figure S4), and a 29%–44% yield decrease relative to the control across the three field seasons (Table 1). Thinner leaves and stems were also noted in T14 in the greenhouse study, but unlike in the field, these deficiencies were compensated for by an increase in tiller number (Fu *et al*., 2012). As leaf area influences photosynthetic efficiency and can ultimately affect growth rates and yields (Ishiwata *et al*., 2013; Tsukaya, 2006), it is possible that the narrow leaves, in the absence of an increased tiller number, contributed to the yield decrease observed in field‐grown line T14.

First year data for the moderate‐expressing transgenic lines were generally consistent with the greenhouse findings, with both lines exhibiting two‐ to fivefold increases in tiller number over the control, and biomass yields either equal to (T27) or greater than (T37) the control. However, the increased tillering phenotypes were lost after year two, and considerable yield reductions were observed in both lines by year three. Line T37, which produced 149% more biomass than the control in the first season, produced 40% less biomass in year three. Yield reductions were most severe in line T27, which produced 80%–96% less biomass than the control in years two and three. The short phenotype of T27 appeared to be detrimental to its growth across multiple years in the field, during which it became progressively shaded each season by neighbouring plants (Figure S5). Excessive shading can cause a reduction in tillering in grasses (Evers *et al*., 2006; Pierson *et al*., 1990), which might explain the tiller reduction in observed in year three. The high susceptibility of T27 to *Bipolaris* (Figure 3) likely further contributed to its stunted growth.

Relative miR156 expression levels within transgenic lines were generally stable between greenhouse and field‐grown lines and across multiple seasons the field. Likewise, the repression of *PvSPL* genes was generally consistent within transgenic lines over the 3‐year study (Figures S2 and S3). Despite this, there was significant year‐to‐year variability in growth within transgenic lines. The inconsistent phenotypes suggest that environmental effects might be overcoming some of the positive growth effects (i.e. increased tiller number) associated with overexpression of the transgene. Our results suggest that transgenic plants with modifications to transcriptional regulators, which impact such a broad range of genes, may require more extensive field evaluations than those with single‐gene modifications to evaluate the stability of the desired growth phenotypes.

### Flowering

Pollen‐mediated gene flow from transgenic feedstocks into native plant populations is an important regulatory concern associated with the implementation of genetically modified plants in agriculture (Kausch *et al*., 2010). Delaying or preventing flowering in transgenic plants is a potential bioconfinement strategy to prevent transgene escape. In the greenhouse study, lines with low miR156 expression levels flowered normally, while those with moderate levels were reported to be nonflowering (Fu *et al*., 2012). As expected, low‐expressing lines T14 and T35 produced flowers each field season, while moderate‐expressing line T27 did not flower for the duration of the study. However, the other moderate‐expressing line (T37) did produce flowers in the field. These results suggest that the ability of miR156‐overexpressing switchgrass lines to flower is not only influenced by the relative level of transgene expression, but also the growth environment. In order for miR156 overexpression to be considered a viable approach for transgene containment in switchgrass, it will be important to first assess their growth across a wide range of field environments.

### Cell wall chemistry and recalcitrance

The presence of lignin in the cell wall limits the accessibility of cell wall sugars for enzymatic hydrolysis and downstream fermentation into biofuel (Chen and Dixon, 2007; DeMartini *et al*., 2013; Himmel *et al*., 2007). Given its role in recalcitrance, it is relevant to examine potential effects of genetic modifications on lignin biosynthesis. In the greenhouse study, only the transgenic lines with high miR156 levels had reductions in lignin content, while the lignin contents of the moderate and low miR156‐expressing lines were similar to the control (Fu *et al*., 2012). In the field, minor or negligible effects on lignin were observed in low miR156‐expressing lines T14 and T35, while lignin was clearly affected in moderate miR156‐expressing lines T27 and T37. Most notably, a 9%–21% reduction in the S/G ratio in these two lines was observed across all three seasons (Tables 2 and S2). A recent study showed that the suppression of the miR156‐targeted *PvSPL2* gene led to a reduction in lignin content and altered lignin composition in transgenic switchgrass lines (Wu *et al*., 2016). In line with these findings, lignin content and composition were primarily affected in the two lines (T27 and T37) that showed the largest reductions in *PvSPL2* expression relative to the other lines and the control (Figures S2 and S3).

Reducing cell wall recalcitrance in switchgrass confers improvements in sugar release and/or bioconversion efficiency into ethanol (Fu *et al*., 2011a,b; Saathoff *et al*., 2011; Shen *et al*., 2013; Xu *et al*., 2011). A previous study found that switchgrass lines overexpressing the maize *Corngrass1* miR156 had an increase in starch content, which was accompanied by an improvement in saccharification efficiency (Chuck *et al*., 2011). In the study by Fu *et al*. (2012), miR156‐overexpressing switchgrass lines grown under greenhouse conditions were less recalcitrant to enzymatic hydrolysis when no pretreatment was performed, but this effect was lost in many lines when a dilute acid pretreatment was performed (Fu *et al*., 2012). In the field, improvements in sugar release were observed intermittently in lines T27 and T37. The two lines (T35 and T37) selected for bioconversion analysis produced more ethanol than the control in year one, but no differences were observed in the two subsequent seasons (Table 1). In agreement with the conclusions of the greenhouse study (Fu *et al*., 2012), the effects of miR156 overexpression on the recalcitrance of these lines when grown in the field were inconclusive, but it appears that there are no negative impacts.

### Disease susceptibility

Rust caused by *P*. *emaculata* and leaf spot caused by *Bipolaris* species have been identified as prevalent fungal pathogens of switchgrass (Krupinsky *et al*., 2004; Uppalapati *et al*., 2013; Vu *et al*., 2011, 2013; Zale *et al*., 2008). Such pathogens could pose serious threats to switchgrass when grown in large‐scale monocultures for biofuel production (Stewart and Cromey, 2011). Consequently, an indispensable component of field experiments with transgenic lines is to ensure that plant defences are not compromised by their genetic modifications. Previous studies showed that modifications to the lignin biosynthetic pathway in transgenic switchgrass had no adverse effects on rust susceptibility across two‐ to 3‐year timespans in the field (Baxter *et al*., 2014, 2015, 2016). However, in contrast to modifications targeting a specific biosynthetic pathway (e.g. lignin), master regulators like microRNAs have a much broader range of downstream target genes. Therefore, possible pleiotropic effects associated with their manipulation could be more extensive. In the current study, rust disease severity was rated across the second and third growing seasons and *Bipolaris* incidence was monitored. Low miR156‐expressing lines T14 and T35 had a similar degree of rust infection as the control and minor symptoms of *Bipolaris* (Figures 2 and 3), while moderate miR156‐expressing lines T27 and T37 both showed altered disease susceptibilities. In line T37, the severity of rust infection changed dramatically between the two subsequent field seasons: susceptibility was lower than the control in 2014, but higher in the following season. An interesting result was also observed in line T27, which exhibited very minor or no rust symptoms across the experiment (Figure 2). Similarly, a previous 2‐year field study showed that a switchgrass line with a relatively high level of MYB4 overexpression did not show any symptoms of rust, whereas susceptibilities of the transgenic lines with relatively lower transgene expression levels were comparable to the control (Baxter *et al*., 2015). High levels of constitutive transgene overexpression might be more likely to cause pleiotropic effects on defence‐related pathways or alter the chemical composition of the plant in such a way to make it a less suitable host for certain pathogens. As there was little to no apparent competition from the rust pathogen in T27, this could have enabled *Bipolaris* to become more virulent in this line relative to those showing normal or high levels of rust infection.

## Conclusions

Genetic engineering has enabled significant progress towards identifying, understanding and successfully manipulating important pathways involved in plant growth and development. Increasing miR156 expression appears to be associated with several desirable bioenergy traits in plants including increased biomass yields. However, our understanding of how yield‐related modifications in greenhouse‐grown plants translate into improved crop performance under realistic agricultural conditions is limited. The findings of our study emphasize that greenhouse results, while providing valuable data on potentially useful targets for yield improvement, must be interpreted with caution until validated under appropriate field environments. The stability of growth‐related traits in transgenic plants, especially those with modified regulatory genes, will likely depend strongly on the environment in which they are grown. Multiyear and multisite field trials across locations that are within a region for targeted cultivation will be critical for evaluating such crops. In addition, as we have noted with another master regulatory gene, the MYB4 transcription factor (Baxter *et al*., 2015), tuning expression for miRNAs and other regulatory genes will be crucial to maximize desired phenotypes in bioenergy feedstocks.

## Experimental procedures

### Agronomic performance

A complete randomized design was used in which all four transgenic lines and a control line, field‐transplanted in early summer 2013, were each replicated in four plots, each containing four clones (Figure S6; see Appendix S1 for more detail). Growth measurements were recorded in August (mid‐season) and December (end‐of‐season) of each field growing season (Figure S7). Mid‐season measurements included tiller height and plant width. End‐of‐season measurements included tiller height, plant width, tiller number, and total aboveground dry weight yield. Growth parameters were measured as described previously (Baxter *et al*., 2014). Briefly, tiller height (measured on the tallest tiller) and whole plant width were measured on all four clonal plants within each replicate. For the end‐of‐season biomass yield, the four clones within each replicate were pooled to represent the total yield of each replicate. Dry weight yield was determined after drying biomass in an oven (43 °C, approximately 168 h).

### *miR156* transgene expression

Samples for quantitative reverse transcription polymerase chain reaction (qRT‐PCR) analysis were collected from plants at the same date and time in August of each growing season. Randomly selected R1‐stage tillers from each replicate were cut from below the top node and the top portion (with top two leaves intact), flash frozen in liquid nitrogen and stored in −80 °C. Total RNA was extracted from the frozen tissues by Tri‐Reagent (Life Technologies, Carlsbad, CA). The transcript abundance of OsmiR156 and PvSPL1, 2, 3 and 6 was analysed as described previously (Fu *et al*., 2012). The mature miR156 level was quantified using a modified method from the stem‐loop RT‐PCR procedure. Briefly, 5 μg of RNA from each samples was mixed with 2 μL of 10 mm dNTPs; then, H_2_O was added to bring final volume to 20 μL. Each mixture was equally split into two parts. In one part, 1 μL of miR156‐specific reverse stem‐loop transcription primer (50 μm) was added with Superscript III reverse transcriptase (Life Technologies) to measure miR156 expression as described (Cui *et al*., 2014). In the other part, 1 μL of 50 μm oligo(dT)_20_ was added to synthesize the total cDNA, which was used to measure switchgrass ubiquitin 1 (*Ubi1*) transcripts (GeneBank accession number: FL899020). The expression data of *miR156* were normalized to that of *Ubi*. SYBR green (Life Technologies) was used as reporter dye. The qRT‐PCR was carried out using ABI PRISM 7900 HT system (Applied Biosystems, Foster City, CA). Oligonucleotide primers are listed in Table S3.

### Cell wall characterization

Mid‐season whole tiller samples (green) were collected in August of each year, and end‐of‐season (senesced) samples were collected each December. Green R1 developmental stage tillers (Hardin *et al*., 2013) were collected from each of the four clonal plants within each replicate and pooled to represent a single biological replicate and then oven dried at 43 °C for 72 h prior to milling with a Wiley mill (Model 4; Thomas Scientific, Swedesboro, NJ) through a 1‐mm screen. For the senesced end‐of‐season samples, subsamples for each replicate were taken from the final harvest of aboveground material after oven‐drying and weighing for total biomass yield. Senesced subsamples were chipped into 5–8 cm pieces prior to milling through a 1‐mm screen.

Pyrolysis mass beam mass spectrometry was performed to analyse cell wall lignin content and the S/G ratio following published methods (Sykes *et al*., 2009). Prior to the analyses, extractives‐ and starch‐free cell wall residues (CWR) were prepared by treating biomass samples with amylases followed by an ethanol extraction in a Soxhlet exctractor (Decker *et al*., 2012). Approximately 4 mg of CWR was pyrolysed at 500 °C in 80 stainless steel cups using an Extrel single quadrupole molecular beam mass spectrometer. Lignin content was estimated as the sum of the intensities of lignin precursor peaks. The S/G ratio was estimated as the intensity of the syringyl peaks divided by the intensity of the guaiacyl peaks.

Sugar release by enzymatic hydrolysis was determined on hot water‐pretreated CWR (prepared as described above; Decker *et al*., 2012) using the high‐throughput method described by Selig *et al*. (2010). In brief, CWR were loaded into a custom‐made 96‐well metal plates in triplicate. Pretreatment was performed with condensing steam (180 °C, 17.5 min). Samples were then subjected to enzymatic hydrolysis by incubation with 70 mg protein/g biomass Ctec2 enzyme cocktail (Novozymes North America, Franklinton, NC) at 50 °C for 70 h. Concentrations of glucose and xylose released were measured using the D‐Glucose Assay Kit (glucose oxidase/peroxidase; GOPOD) and D‐Xylose Assay Kit (xylose dehydrogenase; XDH) (Megazyme Intl., Bray, Ireland).

### Ethanol yield analysis

Ethanol yields were measured by separate hydrolysis and fermentation (SHF) as described previously (Dumitrache *et al*., 2016). SHF experiments were performed in biological duplicate at a 5.0% (w/v) dry solids loading at 20 mL final volume. Biomass samples were incubated at 50 °C for 5 days with hydrolysing enzymes Cellic® Ctec2 (24 FPU/g cellulose), Novozyme 188 (loaded at a 25% volume ratio to Ctec2) and Cellic^®^ Htec2 (loaded at a 20% volume ratio to Ctec2), along with 0.1 mm streptomycin. Enzymes were provided by Novozymes North America (Franklinton, NC) and Sigma‐Aldrich (St. Louis, MO). Following enzymatic hydrolysis, the resulting sugars were fermented at 35 °C for 72 h with *S. cerevisiae* D5α (ATCC 200062) supplemented with yeast extract at 0.5% w/v and 50 mm citrate buffer. No biomass controls of the SHF process were used as baseline measurements accordingly. End point ethanol yield was measured against known standards by HPLC using an Aminex™ Hercules, CA HPX‐87H column (Bio‐Rad Laboratories Inc., CA) at 60 °C and 5 mm H_2_SO_4_ mobile phase at 0.5 mL/min flow rate.

### Rust disease assessment

The susceptibility of switchgrass plants to rust infection was assayed at weekly time points between July and August of 2014 and 2015 as described previously (Baxter *et al*., 2014). Briefly, single tillers from two clonal plants within each replicate plot were randomly selected and tagged, and all leaves along each selected tiller were examined for rust severity at each time point. The coverage of the top leaf surface with rust uredia was visually assessed using the following scale: 0 = 0%, 1 ≤ 5%, 2 ≤ 10%, 3 ≤ 25%, 4 ≤ 40%, 5 ≤ 55%, 6 ≤ 70%, and 7 ≤ 100% of leaf area coverage with uredia. Because of the severity of the rust, the entire field site was sprayed with fungicide in late August of each growing season. All data reported were collected prior to fungicide treatments. Fungicides used included “Quilt” (Syngenta Canada Inc., Guelph, Ontario) at a rate of 0.21 mL/m^2^, and “Heritage” (Syngenta Crop Protection, Greensboro, NC) at a rate of 20 mL/m^2^.

### Statistics

Statistical analyses were performed in SAS version 9.4 (SAS Institute Inc., Cary, NC). Within each year, a one‐way ANOVA with Fisher's least significant difference method was used to compare means among transgenic lines and the control. Differences were considered significant where *P*‐values were less than or equal to 0.05.

## Supporting information


**Figure S1** Relative expression of OsmiR156b in R1 tillers of transgenic switchgrass plants as determined by quantitative RT‐PCR.
**Figure S2** Relative expression of (a) *PvSPL1*, (b) *PvSPL2*, (c) *PvSPL3*, and (d) *PvSPL6* genes in R1 tillers of transgenic switchgrass plants as determined by quantitative RT‐PCR.
**Figure S3** Relative expression of (a) *PvSPL1*, (b) *PvSPL2*, (c) *PvSPL3*, and (d) *PvSPL6* genes in R1 tillers of transgenic switchgrass plants as determined by quantitative RT‐PCR. Samples were harvested in year three (2015) of the field experiment on August 31, 2015.
**Figure S4** Visual comparison of the morphological differences between low‐expressing transgenic line T14 and the wild‐type (WT) control.
**Figure S5** Photos of miR156‐overexpressing transgenic line T27 growing in the field on (a) September 27 2013, (b) September 17 2014, and (c) August 26 2015.
**Figure S6** Field design for evaluation of miR156‐overexpressing switchgrass lines.
**Figure S7** Photos of the field experiment with miR156‐overexpressing switchgrass during the first (2013), second (2014), and third (2015) growing seasons. (a) September 27, 2013; (b) November 25, 2013; (c) August 1, 2014; (d) November 12, 2014; (e) August 10, 2015; (f) December 8, 2015.
**Table S1** Mid‐season morphology of miR156‐overexpressing switchgrass in the first (2013), second (2013), and third (2015) field growing seasons.
**Table S2** Cell wall characterization of miR156‐overexpressing switchgrass harvested in the middle of the growing season in years one (2013), two (2014), and three (2015) of the experiment
**Table S3** Primers used in this study.
**Appendix S1** Supplementary experimental procedures. Plants and field design.
