# Supplementary material for: Transgenic miR156 switchgrass in the field: growth, recalcitrance and rust susceptibility
Source: Plant Biotechnol J. 2017 Jun 20;16(1):39–49. doi: 10.1111/pbi.12747 (PMC5785337; doi:10.1111/pbi.12747)
Supplement: Supplementary file 1 — Figure S1 Relative expression of OsmiR156b in R1 tillers of transgenic switchgrass plants as determined by quantitative RT‐PCR. Figure S2 Relative expression of (a) PvSPL1, (b) PvSPL2, (c) PvSPL3, and (d) PvSPL6 genes in R1 tillers of transgenic switchgrass plants as determined by quantitative RT‐PCR. Figure S3 Relative expression of (a) PvSPL1, (b) PvSPL2, (c) PvSPL3, and (d) PvSPL6 genes in R1 tillers of transgenic switchgrass plants as determined by quantitative RT‐PCR. Samples were harvested in year three (2015) of the field experiment on August 31, 2015. Figure S4 Visual comparison of the morphological differences between low‐expressing transgenic line T14 and the wild‐type (WT) control. Figure S5 Photos of miR156‐overexpressing transgenic line T27 growing in the field on (a) September 27 2013, (b) September 17 2014, and (c) August 26 2015. Figure S6 Field design for evaluation of miR156‐overexpressing switchgrass lines. Figure S7 Photos of the field experiment with miR156‐overexpressing switchgrass during the first (2013), second (2014), and third (2015) growing seasons. (a) September 27, 2013; (b) November 25, 2013; (c) August 1, 2014; (d) November 12, 2014; (e) August 10, 2015; (f) December 8, 2015. Table S1 Mid‐season morphology of miR156‐overexpressing switchgrass in the first (2013), second (2013), and third (2015) field growing seasons. Table S2 Cell wall characterization of miR156‐overexpressing switchgrass harvested in the middle of the growing season in years one (2013), two (2014), and three (2015) of the experiment Table S3 Primers used in this study. Appendix S1 Supplementary experimental procedures. Plants and field design. [file PBI-16-39-s001.docx]

**Supplementary Materials**

**Appendix S1. Supplementary experimental procedures**

Plants and field design

Switchgrass plants with constitutive overexpression of a rice miR156 were produced in the ‘Alamo’ cultivar background as described previously (Fu et al., 2012). Four independent T0 transgenic events (T14, T35, T27, and T37) and one Alamo wild-type control from this study were used in the current field study. Four replicates for each transgenic event and the control were transplanted to a 14.5 m x 12.2 m field site on a University of Tennessee-Knoxville farm in July of 2013. Replicates were arranged in a completely randomized design. Each transgenic and control replicate plot contained four vegetatively propagated clones. Replicate plots were 152 cm apart with 76 cm spacing among the four clonal plants within each replicate. Experimental plants were surrounded by a border of wild-type plants in the ‘Alamo’-derived ST1 genetic background (Figure S6).

Plants were maintained in the field across three growing seasons (Figure S7). Irrigation was used during the initial growing season to promote successful establishment of transplants. No fertilizer or herbicides were applied for the duration of the study, and weeding was performed by hand or tillage. First flowering occurred in late June or early July of each season. Following USDA APHIS BRS release into the environment permit requirements, panicles of the first reproductive (R1) stage (Hardin et al., 2013) were removed from all plants (transgenic, non-transgenic wild-type control, and border plants) by cutting the tiller from below the top node.

**Supplementary figures**

**
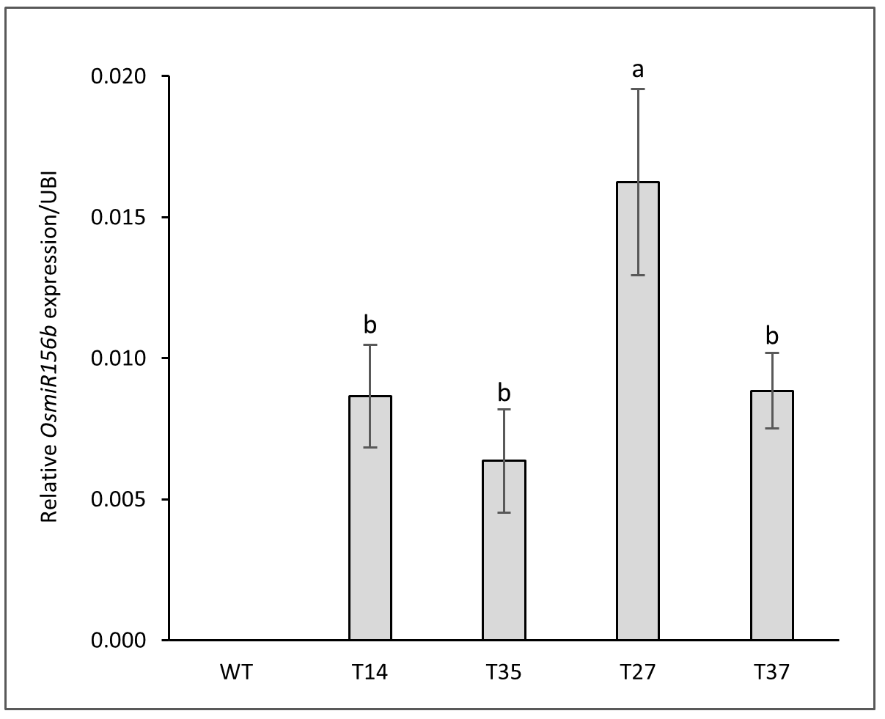
**

**Figure S1.** Relative expression of OsmiR156b in R1 tillers of transgenic switchgrass plants as determined by quantitative RT-PCR. Samples were harvested in year one (2013) of the field experiment on September 10, 2013. The relative levels of transcripts were normalized to ubiquitin (UBI). Bars represent the mean of the biological replicates (n=4) for each low-expressing (T14, T35) and moderate-expressing (T27, T37) transgenic line and the wild-type control (WT) ± standard error. Means were compared with a one-way ANOVA and letter groupings were obtained using Fisher’s least significant difference method. Bars with different letters are significantly different at the 5% level.

**
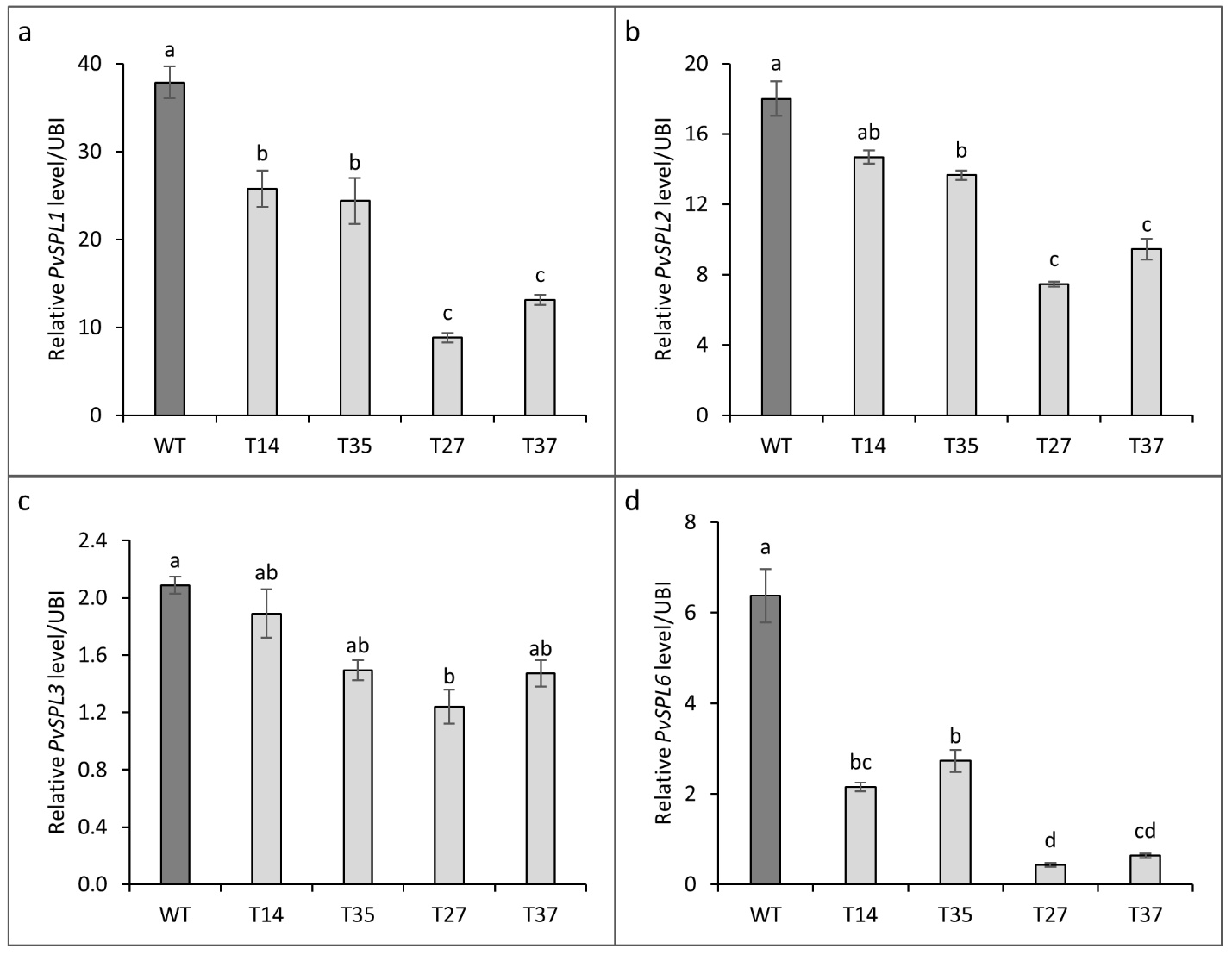
Figure S2.** Relative expression of (a) *PvSPL1*, (b) *PvSPL2*, (c) *PvSPL3*, and (d) *PvSPL6* genes in R1 tillers of transgenic switchgrass plants as determined by quantitative RT-PCR. Samples were harvested in year one (2013) of the field experiment on September 10, 2013. The relative levels of transcripts were normalized to ubiquitin (UBI). Bars represent the mean of the biological replicates (n=4) for each low-expressing (T14, T35) and moderate-expressing (T27, T37) transgenic line and the wild-type control (WT) ± standard error. Means were compared with a one-way ANOVA and letter groupings were obtained using Fisher’s least significant difference method. Bars with different letters are significantly different at the 5% level.

**
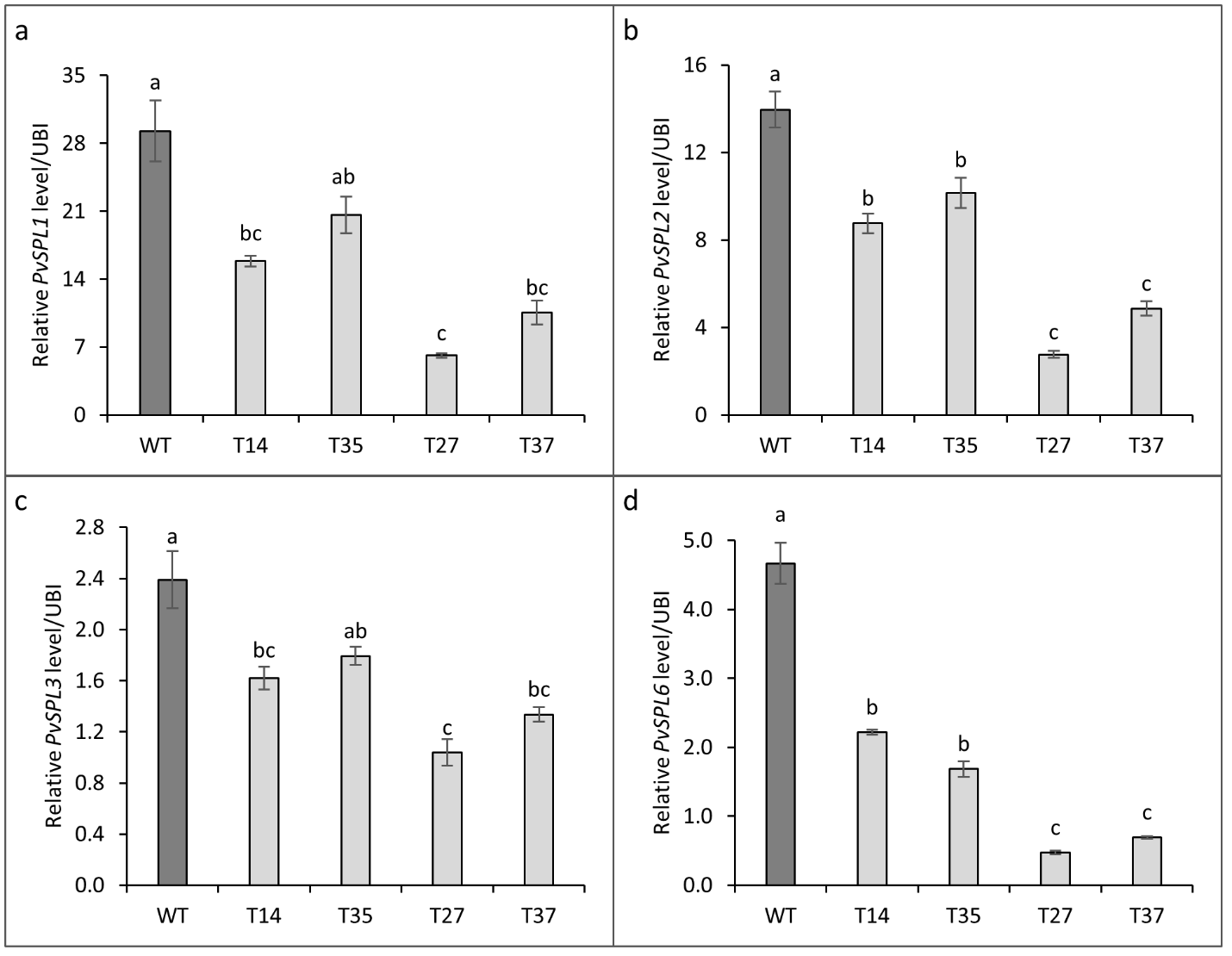
Figure S3.** Relative expression of (a) *PvSPL1*, (b) *PvSPL2*, (c) *PvSPL3*, and (d) *PvSPL6* genes in R1 tillers of transgenic switchgrass plants as determined by quantitative RT-PCR. Samples were harvested in year three (2015) of the field experiment on August 31, 2015. The relative levels of transcripts were normalized to ubiquitin (UBI). Bars represent the mean of the biological replicates (n=4) for each low-expressing (T14, T35) and moderate-expressing (T27, T37) transgenic line and the wild-type control (WT) ± standard error. Means were compared with a one-way ANOVA and letter groupings were obtained using Fisher’s least significant difference method. Bars with different letters are significantly different at the 5% level.


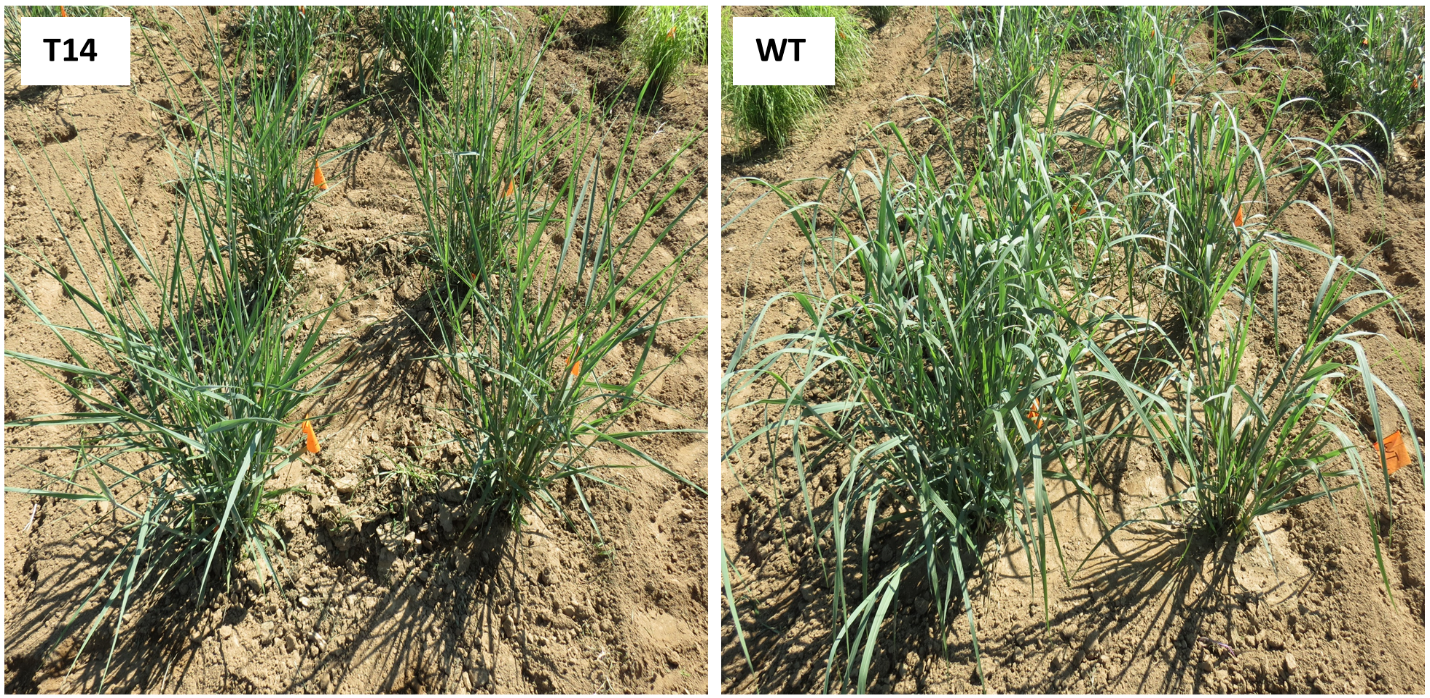


**Figure S4.** Visual comparison of the morphological differences between low-expressing transgenic line T14 and the wild-type (WT) control. The stems of line T14 were thinner and the leaves were narrower and more erect than in the WT control. Photos were taken on October 1, 2013.

**
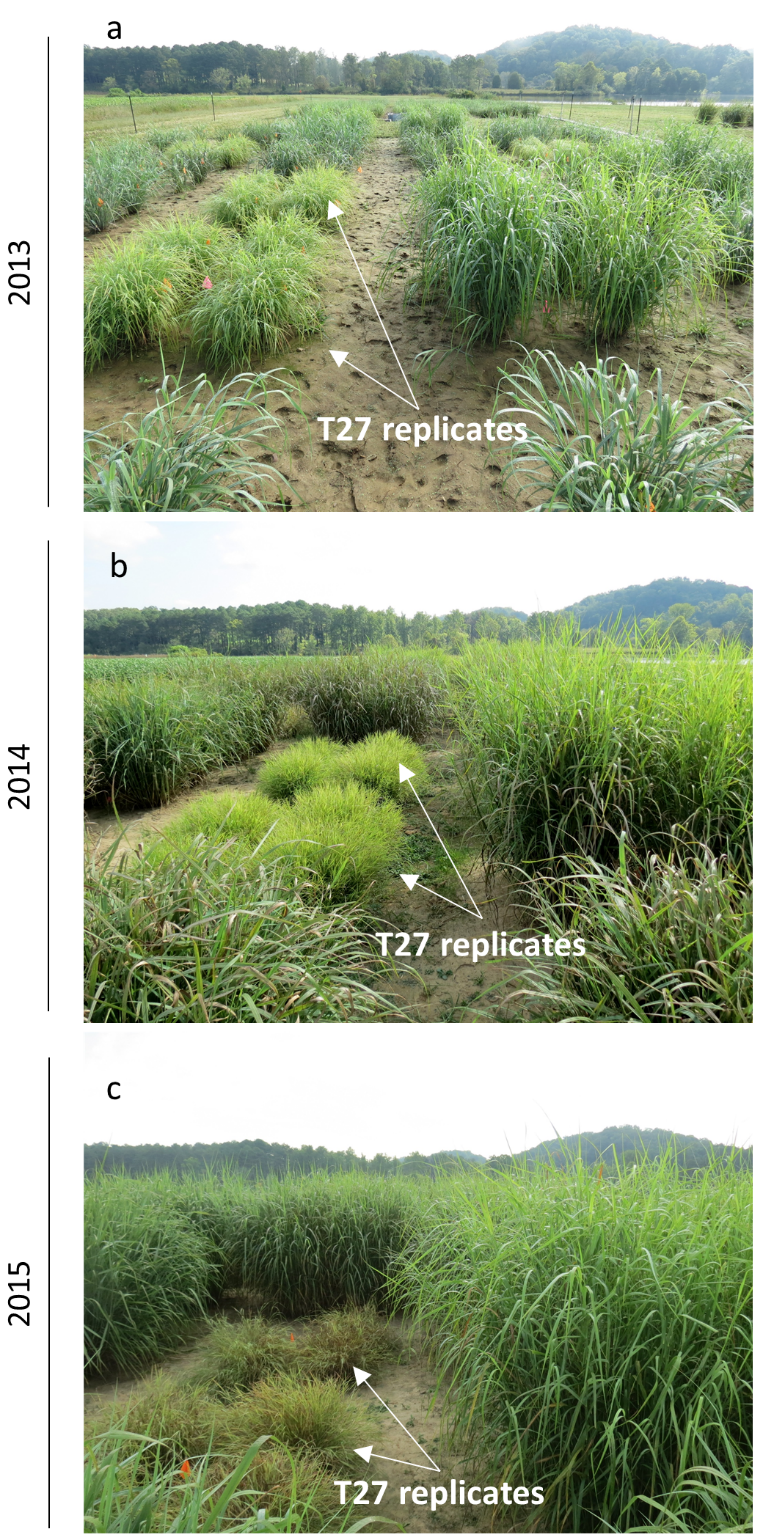
**

**Figure S5.** Photos of miR156-overexpressing transgenic line T27 growing in the field on (a) September 27 2013, (b) September 17 2014, and (c) August 26 2015.

**
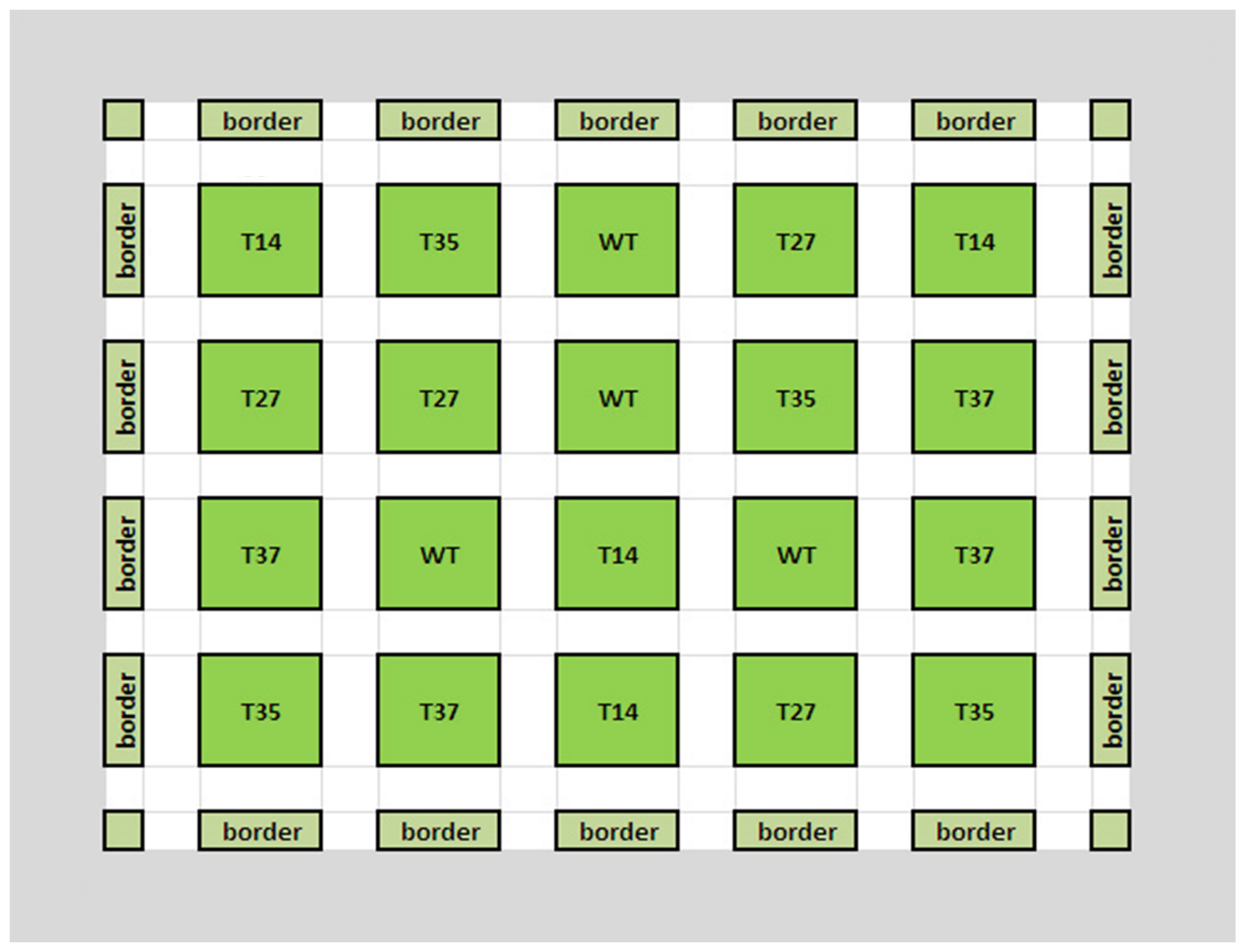
**

**Figure S6.** Field design for evaluation of miR156-overexpressing switchgrass lines. The field site was a 14.5 m x 12.1 m plot. Transgenic (T14, T35, T27, and T37) and wild-type control (WT) replicates were arranged in a completely randomized design and surrounded by a row of non-transgenic ‘Alamo’ border plants to reduced shading effects. Each replicate contained four vegetatively propagated clones of a single transgenic event. Replicates were 152 cm apart with 76 cm spacing among the four clones.


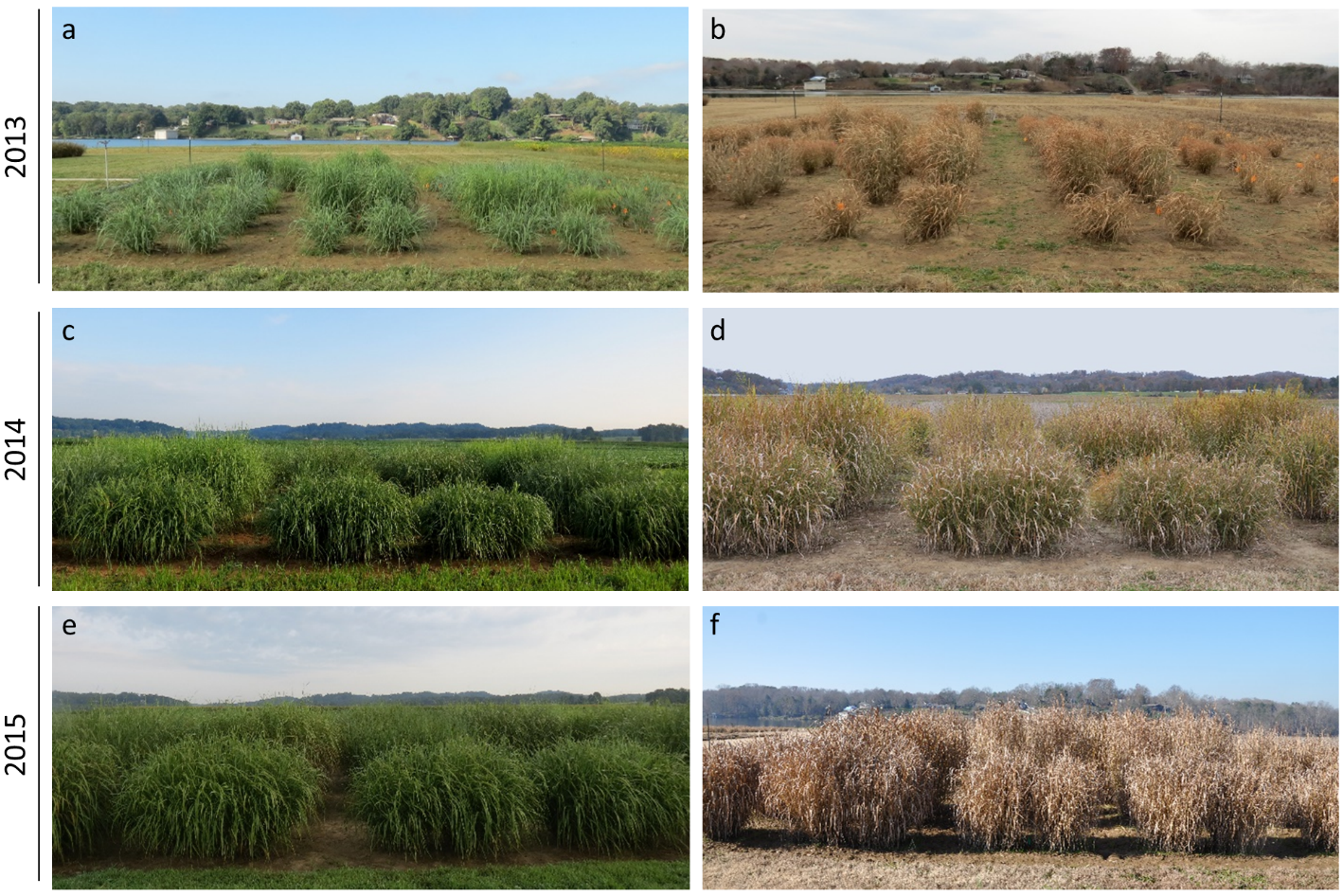


**Figure S7.** Photos of the field experiment with miR156-overexpressing switchgrass during the first (2013), second (2014), and third (2015) growing seasons. (a) September 27, 2013; (b) November 25, 2013; (c) August 1, 2014; (d) November 12, 2014; (e) August 10, 2015; (f) December 8, 2015.

**Supplementary tables**

**Table S1.** Mid-season morphology of miR156-overexpressing switchgrass in the first (2013), second (2013), and third (2015) field growing seasons.

| Year | Line | Tiller height (cm) | | Plant width  (cm) | |
| --- | --- | --- | --- | --- | --- |
| 2013 | T14 | 99.2 ± | 2.1^c^ | 94.1 ± | 2.1^b^ |
|  | T35 | 141.9 ± | 3.6^a^ | 136.2 ± | 4.7^a^ |
|  | T27 | 73.5 ± | 2.3^d^ | 120.7 ± | 6.8^a^ |
|  | T37 | 136.2 ± | 2.0^a^ | 132.9 ± | 7.9^a^ |
|  | WT | 122.7 ± | 3.8^b^ | 118.3 ± | 8.5^a^ |
|  |  |  |  |  |  |
| 2014 | T14 | 156.2 ± | 4.5^b^ | 181.6 ± | 8.8^c^ |
|  | T35 | 170.8 ± | 3.6^a^ | 284.8 ± | 17.6^a^ |
|  | T27 | 67.8 ± | 0.8^c^ | 178.4 ± | 10.6^c^ |
|  | T37 | 179.4 ± | 1.9^a^ | 223.5 ± | 8.0^b^ |
|  | WT | 174.0 ± | 4.7^a^ | 249.9 ± | 11.2^ab^ |
|  |  |  |  |  |  |
| 2015 | T14 | 178.7 ± | 3.3^c^ | 274.5 ± | 8.2^c^ |
|  | T35 | 170.8 ± | 3.6^a^ | 284.8 ± | 17.6^a^ |
|  | T27 | 72.9 ± | 1.0^d^ | 156.5 ± | 5.6^d^ |
|  | T37 | 202.1 ± | 1.3^b^ | 260.0 ± | 4.6^c^ |
|  | WT | 215.5 ± | 0.4^a^ | 340.5 ± | 5.3^b^ |

Values represent the mean of the biological replicates (n=4) for each low-expressing (T14, T35) and moderate-expressing (T27, T37) transgenic line and the wild-type control (WT) ± standard error. Means within each year were analyzed with a one-way ANOVA and letter groupings were obtained using Fisher’s least significant difference method. Values followed by different letters are significantly different at the 5% level.

**Table S2.** Cell wall characterization of miR156-overexpressing switchgrass harvested in the middle of the growing season in years one (2013), two (2014), and three (2015) of the experiment.

| Year | Line | Lignin content  (% CWR) | | S/G ratio | | Glucose release (mg/g CWR) | | Xylose release (mg/g CWR) | | Total sugar release  (g/g CWR) | |
| --- | --- | --- | --- | --- | --- | --- | --- | --- | --- | --- | --- |
| 2013 | T14 | 19.9 ± | 0.2^a^ | 0.57 ± | 0.01^a^ | 0.260 ± | 0.01^b^ | 0.206 ± | 0.01^a^ | 0.466 ± | 0.01^ab^ |
|  | T35 | 19.0 ± | 0.2^ab^ | 0.55 ± | 0.01^a^ | 0.296 ± | 0.01^a^ | 0.207 ± | 0.01^a^ | 0.503 ± | 0.01^a^ |
|  | T27 | 19.8 ± | 0.5^a^ | 0.49 ± | 0.02^c^ | 0.237 ± | 0.01^b^ | 0.207 ± | 0.02^a^ | 0.444 ± | 0.01^b^ |
|  | T37 | 19.9 ± | 0.3^a^ | 0.50 ± | 0.00^bc^ | 0.255 ± | 0.01^b^ | 0.192 ± | 0.00^a^ | 0.447 ± | 0.01^b^ |
|  | WT | 18.4 ± | 0.3^b^ | 0.54 ± | 0.02^ab^ | 0.300 ± | 0.01^a^ | 0.204 ± | 0.00^a^ | 0.504 ± | 0.01^a^ |
|  |  |  |  |  |  |  |  |  |  |  |  |
| 2014 | T14 | 24.7 ± | 0.1^a^ | 0.68 ± | 0.00^a^ | 0.163 ± | 0.01^c^ | 0.190 ± | 0.00^a^ | 0.353 ± | 0.01^c^ |
|  | T35 | 23.0 ± | 0.4^ab^ | 0.64 ± | 0.01^b^ | 0.195 ± | 0.01^b^ | 0.203 ± | 0.01^a^ | 0.398 ± | 0.01^b^ |
|  | T27 | 21.5 ± | 0.5^b^ | 0.54 ± | 0.01^d^ | 0.220 ± | 0.01^a^ | 0.222 ± | 0.01^a^ | 0.443 ± | 0.01^a^ |
|  | T37 | 23.1 ± | 0.5^ab^ | 0.59 ± | 0.01^c^ | 0.185 ± | 0.00^b^ | 0.201 ± | 0.01^a^ | 0.386 ± | 0.01^b^ |
|  | WT | 23.2 ± | 0.5^ab^ | 0.68 ± | 0.01^a^ | 0.177 ± | 0.00^bc^ | 0.198 ± | 0.01^a^ | 0.375 ± | 0.01^bc^ |
|  |  |  |  |  |  |  |  |  |  |  |  |
| 2015 | T14 | 22.6 ± | 0.4^a^ | 0.60 ± | 0.01^a^ | 0.194 ± | 0.01^c^ | 0.198 ± | 0.01^b^ | 0.393 ± | 0.01^b^ |
|  | T35 | 21.7 ± | 0.2^ab^ | 0.60 ± | 0.02^a^ | 0.209 ± | 0.00^bc^ | 0.201 ± | 0.01^b^ | 0.410 ± | 0.01^b^ |
|  | T27 | 19.7 ± | 0.1^c^ | 0.50 ± | 0.01^b^ | 0.238 ± | 0.00^a^ | 0.208 ± | 0.00^b^ | 0.447 ± | 0.01^a^ |
|  | T37 | 21.0 ± | 0.4^b^ | 0.53 ± | 0.01^b^ | 0.226 ± | 0.01^ab^ | 0.226 ± | 0.01^a^ | 0.452 ± | 0.02^a^ |
|  | WT | 21.9 ± | 0.3^a^ | 0.62 ± | 0.01^a^ | 0.197 ± | 0.01^c^ | 0.198 ± | 0.00^b^ | 0.396 ± | 0.01^b^ |

Green tillers of the first reproductive stage (Hardin et al., 2013) were analyzed at mid-season for lignin content, syringyl-to-guaiacyl (S/G) lignin monomer ratio, and sugar release by enzymatic hydrolysis. Values represent the mean of the biological replicates (n=4) for each transgenic line (T14, T35, T27, T37) and the wild-type control (WT) ± standard error. Means within each year were compared with a one-way ANOVA and letter groupings were obtained using Fisher’s least significant difference method. Values followed by different letters are significantly different at the 5% level. CWR, cell wall residues.

**Table S3.** Primers used in this study.

| OsmiR156-F | CACCACAGTTTAATTTATTTCTTGG |
| --- | --- |
| OsmiR156-R | CTAGGCAGAAAATTTAACAGGAG |
| miR156 Stem-loop primer | CGCGAGCTCAGAATTAATACGACTCACTATACGCGGTGCTC |
| miR156-F | GGCGGTGACAGAAGAGAGT |
| miR Stem-R | CGCGAGCTCAGAATTAATACGA |
| PvSPL1-F | GATGGCCTGGGGTCTTG |
| PvSPL1-R | TGGCCTACGCTCAGTTC |
| PvSPL2-F | GCGCGGTTTCAGGCTCTCG |
| PvSPL2-R | CCTCGCAACCGGACAATGGA |
| PvSPL3-F | GCCACGCCACGACCACGAGAC |
| PvSPL3-R | CGGGCTACACGGGAAAGGGGAACT |
| PvSPL6-F | GCAGCGGCGGCGACCAGGAC |
| PvSPL6-R | GGCAGATCGACAGACACAGCACAC |
| PvUbi-F | TTCGTGGTGGCCAGTAAGC |
| PvUbi-R | AGAGACCAGAAGACCCAGGTACAG |
